# Hemorrhagic Cholecystitis in a Patient Under Anticoagulant and Antiplatelet Therapy: A Case Report

**DOI:** 10.7759/cureus.60378

**Published:** 2024-05-15

**Authors:** Eduardo Ichikawa-Escamilla, Xiomara G Solís-Ibarra, Zuleyma Lizárraga-López, Rafael Valenzuela-Hernández, José A Flores-Díaz

**Affiliations:** 1 Surgery, Hospital General de Zona No 14 con Unidad de Quemados, Instituto Mexicano del Seguro Social, Hermosillo, MEX

**Keywords:** hemorrhagic cholecystitis, perforated gallbladder, anticoagulant therapy, antiplatelet therapy, subtotal cholecystectomy

## Abstract

Hemorrhagic cholecystitis is an uncommon presentation of acute cholecystitis. Due to its etiology and unspecific clinical data, it is an entity that represents a diagnostic challenge. We present a case of a 70-year-old male with diabetes type 2, hypertension, and chronic kidney disease with hemodialysis, who attended the emergency department with sudden-onset abdominal pain in the epigastrium. The patient presented no additional symptoms, a normal electrocardiogram, but due to the characteristics of the pain and elevated troponin I, emergency medicine specialists considered an acute coronary syndrome and initiated antiplatelet and anticoagulant therapy. Due to persistent abdominal pain, a decrease in hemoglobin, and the onset of arterial hypotension, a computed tomography (CT) scan was performed, which revealed perforation of the gallbladder, apparent hemorrhagic cholecystitis, and hemoperitoneum. The patient underwent emergent surgery, where CT findings were confirmed. In our case, the suspicion of hemorrhagic cholecystitis arose until the clinical case was advanced, after receiving anticoagulant and antiplatelet therapy, and it was confirmed during surgery and with histopathology. This concludes that hemorrhagic cholecystitis is a rare disease and difficult to diagnose. Therefore, studies should focus on clinical presentation and risk factors (e.g., trauma, malignancy, renal failure, cirrhosis, and anticoagulation therapy) to promote early diagnosis and avoid complications.

## Introduction

Hemorrhagic cholecystitis is a rare complication of acute cholecystitis, and it is defined as hemorrhage inside the gallbladder [1]. This condition has been associated with uremia, renal disease, atrial fibrillation, anticoagulant or antiplatelet therapy, chronic obstructive pulmonary disease, chronic use of corticosteroids, cirrhosis, and blunt trauma [2,3]. Hemorrhagic cholecystitis is presented at an average age of 65 years, and 70% of cases reported were men [3].

Symptoms of hemorrhagic cholecystitis include right hypochondrium pain, fever, and jaundice. Only 21% of the patients present hemorrhagic signs, such as hypotension, lethargy, anemia, and melena. Between laboratory alterations, 56% present leukocytosis and 9% hyperbilirubinemia [3]. Abdominal ultrasound (US) and computed tomography (CT) scan are image studies mostly used [4]. A confirmatory study is done with histopathology [3].

The treatment of choice is cholecystectomy; however, the approach is still under discussion. Urgent surgery is ideal to avoid perforation of the gallbladder. The laparoscopic approach can be performed in hemodynamically stable patients and the open approach in unstable patients. Another therapeutic option is cholecystostomy. In the case of subtotal cholecystectomy and postoperative biliary leakage, endoscopic retrograde cholangiopancreatography (ERCP) can be performed as a complementary treatment [3,4].

## Case presentation

A 70-year-old male patient was admitted to the emergency department of our hospital unit due to abdominal pain. As pathological antecedents, he referred to hypertension, diabetes type 2, and chronic kidney disease. On admission, the patient reported a sudden onset of pain, located in the epigastrium, intense and radiating to both hypochondria; he denied respiratory distress, clinical data of low cardiac output, and vegetative symptoms. Physical examination revealed pain on palpation of the epigastrium. Vital signs at admission were heart rate of 85 beats per minute, respiratory rate of 16 breaths per minute, blood pressure of 130/83 mmHg, temperature of 38 degree Celsius, and oxygen saturation of 98%. Laboratory results at admission are demonstrated in Table 1. A 12-lead electrocardiogram was performed, which showed no evidence of ischemia or other significant modifications.

**Table 1:** Laboratories at hospital admission.

Laboratories	Results	Reference ranges
Hemoglobin	10.1 g/dL	12.21–18.1
Hematocrit	27.9%	37.7–53.7
Mean corpuscular volume (MCV)	89 fL	80–97
Platelet count	236 x 10^9^/L	142–424
White blood count	9,000/mm^3^	4,600–10,200
Creatinine	3.6 mg/dL	0.5–1.2
Urea	96.3 mg/dL	16.6–48.5
Blood urea nitrogen (BUN)	45 mg/dL	8–23
Glucose	121 mg/dL	74–106
Creatine phosphokinase (CPK)	57 UI/L	32–294
Creatine phosphokinase-MB (CPK-MB)	35 UI/L	0–25
Troponin I	411.30 pg/mL	< 30
Total bilirubin	0.3 mg/dL	0.3–1.2
Alanine aminotransferase	16 UI/L	0–34
Aspartate aminotransferase	18 UI/L	15–40
Alkaline phosphatase	129 UI/L	35–129
Gamma-glutamyl transferase	70 UI/L	1–30
Prothrombin time (PT)	12.9	10–14
Activated partial thromboplastin time (APTT)	33.2	25.1–36.5
International normalized ratio (INR)	1.13	1.0–1.0

Due to cardiovascular risk factors and alteration of cardiac enzymes, it was considered an atypical presentation of acute coronary syndrome without ST elevation; therefore, the patient was admitted to internal medicine service. An anticoagulant (enoxaparin 60 milligrams subcutaneously every 12 hours) and antiplatelet therapy (aspirin 100 milligrams oral every 24 hours, clopidogrel 75 milligrams oral every 24 hours) were initiated. Subsequently, he was evaluated by the cardiology department, which requested cardiac enzymes control and performed an electrocardiogram and echocardiogram, excluding an acute coronary syndrome.

**Figure 1:**
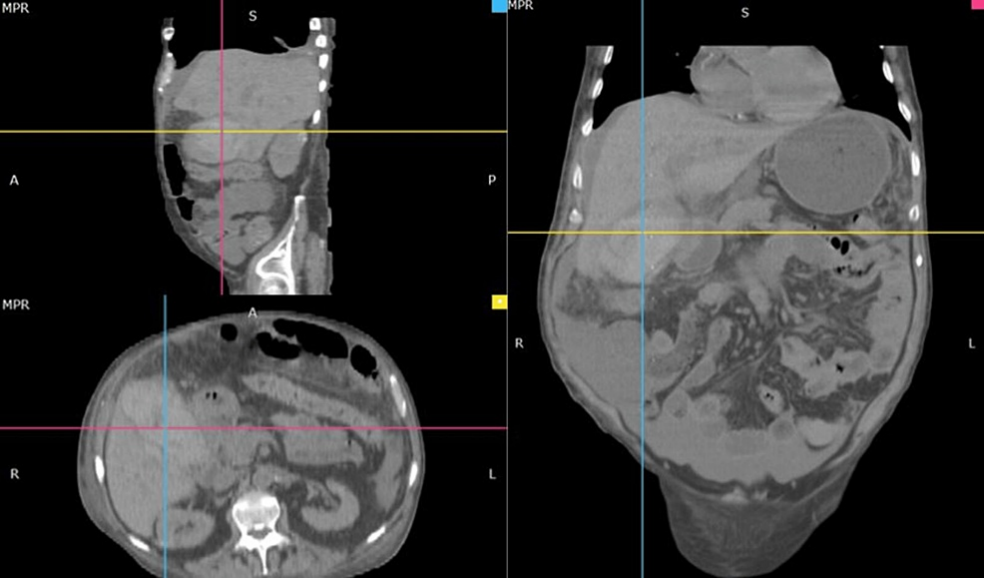
CT of the abdomen and pelvis without contrast. Perforated gallbladder with hematic content.

The patient persisted with generalized pain in all abdominal quadrants, which progressed in intensity. Therefore, control laboratories and a simple abdominal CT were requested, and, afterwords, the general surgery service was asked to evaluate the patient. During the evaluation, the physical examination included data from the acute abdomen (presence of Blumberg's sign, absent peristalsis, abdominal defense, abdominal hypersensitivity), and systemic inflammatory response syndrome (heart rate of 115 beats per minute, respiratory rate of 23 breaths per minute, temperature of 38 degrees Celsius, white blood cell count of 14,100/mm^3^). Hemoglobin control showed anemia grade IV (5.0 g/dL). The CT of abdomen and pelvis revealed overdistended gallbladder, dimensions 125x70x57 mm, containing an average density of 60 Hounsfield units (HU) (probably hematic), with irregular wall, with changes suggesting perforation at the level of the hepatic side of the gallbladder body: abundant free intraperitoneal fluid (Figure 1). Acute abdomen secondary to gallbladder perforation, apparently due to hemorrhagic cholecystitis, was concluded.

**Figure 2:**
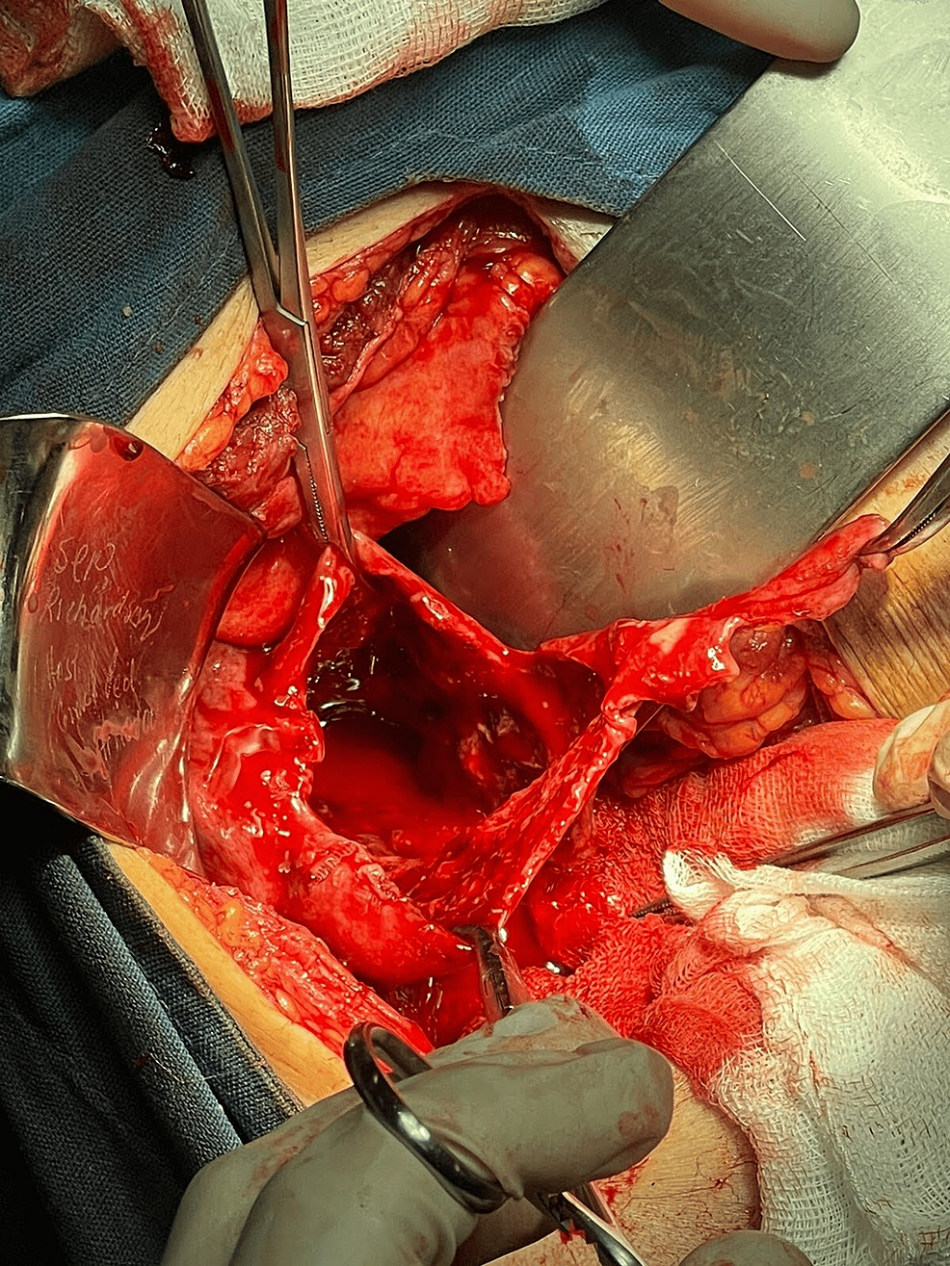
Gallbladder during trans-surgery. Active bleeding, perforation in the fundus, intravesicular clot are visualized.

**Figure 3:**
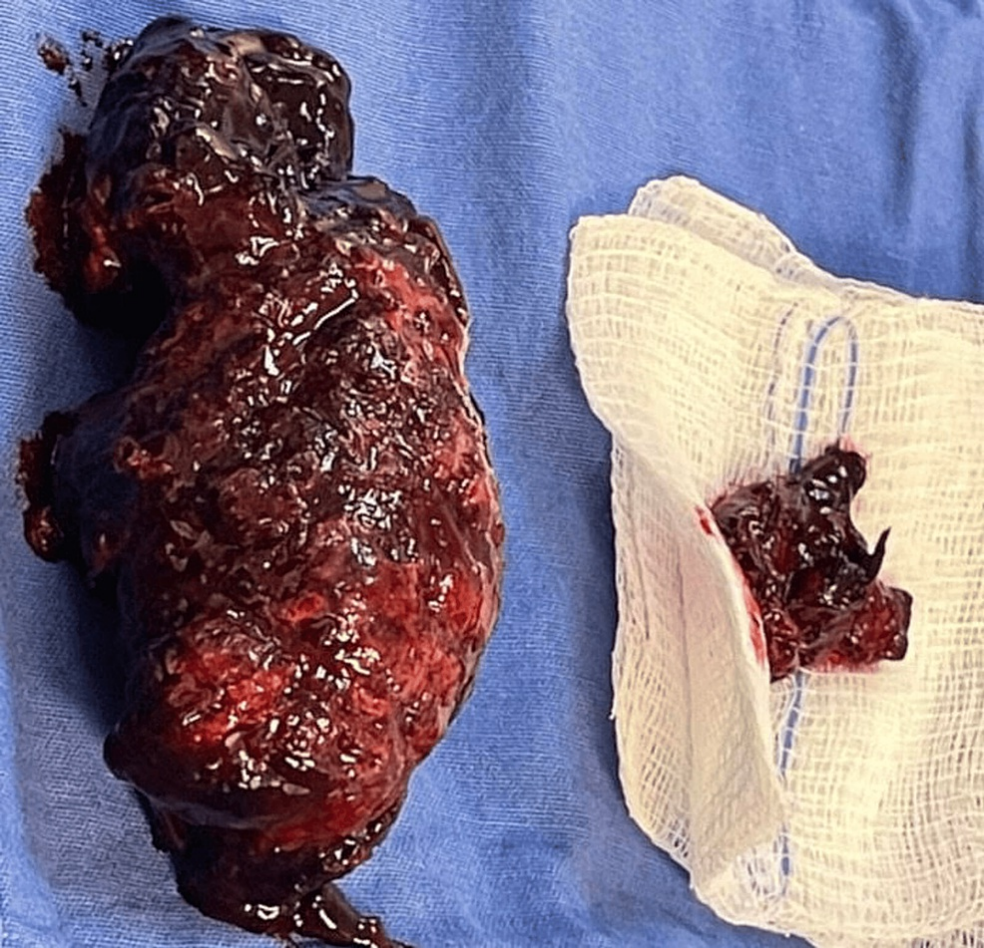
Intravesicular clot.

Transfusion of one pack of red blood cell concentrate was initiated, and following this, the patient underwent emergency open cholecystectomy. During surgery, hemoperitoneum of 1.5 L, perforated gallbladder (Figure 2), with intravesicular clot covering 80% of its interior (Figure 3), active bleeding in the gallbladder wall and in the liver, with trans-surgical bleeding of 0.5 L, was identified. Partial resection of the gallbladder was performed due to difficulty in visualizing Calot's triangle, and an open drainage (Penrose) was placed. The procedure ended as a fenestrated subtotal cholecystectomy. Gallbladder was sent to pathology.

The patient leaves the operating room hemodynamically unstable, dependent on vasopressor amines (norepinephrine), with invasive mechanical ventilation. He was kept under postoperative surveillance, with favorable evolution, and extubated five days later. Because of the presence of a biliary leak in the Penrose drain, ERCP was performed with stent placement, which was removed in three weeks due to the remission of the leak.

The histopathological study of our institution (study number Q1604/2023) reported "vesicular content with fragments of the clot with fibrin, abundant erythrocytes, proteinaceous material, polymorphonuclear, and lymphocytic infiltrate, in addition to the vesicular wall with sphacelated mucosa, with an inflammatory infiltrate of polymorphonuclear, lymphocytes, and plasma cells, accompanied by extravasated erythrocytes that are distributed irregularly in the thickness of the wall." With the above, hemorrhagic cholecystitis was confirmed. Microscopic images are not available.

The patient had an outpatient follow-up with the general surgeon, with adequate postoperative evolution, with no complications associated with the procedure or the initial pathology. Therefore, the patient was definitively discharged from the surgery service.

## Discussion

Currently, the etiology and risk factors associated with hemorrhagic cholecystitis have not been elucidated. In our case, the patient had chronic kidney disease, and due to the suspicion of an acute coronary syndrome, he received anticoagulant and antiplatelet treatment, which could have converted the acute cholecystitis into hemorrhagic, a situation that was demonstrated at surgery and later in the histopathology. It is worth mentioning that the initial inflammation of the gallbladder presents microbleeding in the wall and vascular fragility [5], so antiaggregants and anticoagulants could trigger active intravesicular bleeding. The use of anticoagulant therapy has been previously reported in 45% of patients with hemorrhagic cholecystitis [5]. Few cases of hemorrhagic cholecystitis have been reported in Mexico, and none of them (as far as we know) are specifically associated with anticoagulant and antiplatelet therapy. At last, hemorrhagic cholecystitis is associated with high mortality and morbidity, especially gallbladder perforation and massive hemorrhage [6], which increases the significance of this pathology. Treatment options for hemorrhagic cholecystitis are either cholecystectomy (open or laparoscopy) or cholecystostomy [3]. In our case, open cholecystectomy was performed due to hemodynamic instability, which ended as a fenestrated subtotal cholecystectomy due to the difficulty of visualization of cystic structures.

## Conclusions

Hemorrhagic cholecystitis is a rare pathology, difficult to diagnose, and non-specific clinical presentation, and there is no accurate information on the associated risk factors. In addition, it is associated with high mortality and morbidity. It is important to continue with the research of this disease to establish information with more scientific support and to contribute to an early diagnosis and fewer complications.
